# Differentiating Chilaiditi’s Syndrome with hollow viscus perforation: A case report

**DOI:** 10.1016/j.ijscr.2020.12.029

**Published:** 2020-12-16

**Authors:** Imam Sofii, Zakariya Aji Parminto, Sumadi Lukman Anwar

**Affiliations:** a Digestive Surgery Division, Department of Surgery, Faculty of Medicine, Public Health and Nursing, Universitas Gadjah Mada/Dr. Sardjito Hospital, Yogyakarta, 55281, Indonesia; b Department of Surgery, Faculty of Medicine, Public Health and Nursing, Universitas Gadjah Mada/Dr. Sardjito Hospital, Yogyakarta, 55281, Indonesia; c Oncology Surgery Division, Department of Surgery, Faculty of Medicine, Public Health and Nursing, Universitas Gadjah Mada/Dr. Sardjito Hospital, Yogyakarta, 55281, Indonesia

**Keywords:** Chilaiditi’s syndrome, Chilaiditi’s sign, Pseudoperitoneum, Colonic interposition

## Abstract

**Introduction:**

Chilaiditi‘s syndrome is a rare condition accounting for only 0.25%–0.28% of all abdominal imaging worldwide. To rule out Chilaiditi‘s syndrome from other acute abdominal emergencies is very important to avoid unnecessary treatment or surgical procedure.

**Presentation of case:**

A 25-year-old female presented in the emergency room with 1 week history of abdominal discomfort. At time of examination, she had a mild shortness of breath that was not related with rigorous activities. A plain abdominal x-ray was suggested the presence of an air-filled bowel tract within the right subphrenic space (Fig. 1). Abdominal computed tomography suggested colonic loop present between the right hemi-diaphragm and liver. The absence of abdominal free air confirmed an isolated pseudo-pneumoperitoneum due to colonic interposition between the liver and diaphragm.

**Discussion:**

Chilaiditi sign is radiolucency in the subdiaphragmatic space as a result of bowel interposition between a diaphragm and the liver. If gastrointestinal symptoms present, the condition is known as Chilaiditi's syndrome. The abdominal symptoms including severe pain, anorexia, diarrhea, nausea, vomiting, bloating and constipation might mislead physicians or surgeons with diaphragmatic hernia, subdiaphragmatic abscess, bowel perforation, infected hydatid cyst and liver tumor. Thorough physical examination, imaging, and timely follow up is very important to avoid unnecessary exploratory laparotomies.

**Conclusion:**

Chilaiditi’s Syndrome is often misdiagnosed with bowel perforation because the presence of pseudopneumoperitoneum in the plain X-Rays. It is important to understand the unique characteristics of the sign, symptoms and findings of Chilaiditi’s Syndrome to prevent unnecessary surgical procedures.

## Highlights

• Its important to understand the unique characteristic of the sign, symptoms and findings of Chilaiditi’s Syndrome.
• Chilaiditi’s Syndrome often misdiagnosed with hollow viscus perforation which can lead to unnecessary treatment.
• Conservative treatment has proven to be effective for patient that have Chilaiditi’s Syndrome without any complication.

## 1 Introduction

Chilaiditi syndrome is a rare clinical manifestation in which the signs of acute abdomen or intestinal obstruction are caused by interposition of small or large bowel with the liver and diaphragm. Most patients with Chilaiditi sign of intestinal anomaly are without symptom. However, a small proportion of patients present with intermittent profuse vomiting, abdominal pain or distention, anorexia, diarrhea, and constipation that on very rare conditions need surgery [[1], [2], [3]].

In physiological condition, suspensory ligament of the liver and colon fixation can prevent the bowel interposition with the liver and diaphragm. Anatomical variations including absence, elongation, or laxity of the falciform liver ligament and the suspensory ligament of the transverse colon can predispose the interposition. Congenital malposition and functional disorders including chronic constipation, distension, and elevated intra abdominal pressure can predispose the development of symptoms in the Chilaiditi sign.

Surgical intervention is usually not recommended inpatients with Chilaiditi’s syndrome. However, serious conditions of acute abdomen have to be first ruled out. Intravenous fluid rehydration, abdominal decompression with nasogastric tube, bed rest, and enema or stool softeners are initial management for patients with Chilaiditi’s syndrome. Surgery procedure is only performed if the patient does not respond to the conservative treatment or there is evidence of bowel ischemia or mechanical bowel obstruction. Therefore, differentiating Chilaiditi's syndrome with other acute abdomen as well as careful monitoring during conservative treatment are very important in the management. We reported a case of Chilaiditi's syndrome with severe abdominal pain and responded with conservative treatment following SCARE guidelines [9].

## 2 Presentation of case

A 25-year-old presented to the emergency department in our hospital with 1 week history of abdominal discomfort. His symptoms present with a mild shortness of breath that doesn’t exacerbated with activities. He denied history of fever, nausea & vomiting, melena, constipation or diarrhea. He denied having diabetes mellitus, hypertension, or any history of heart disease. He denied any tobacco use, alcohol, or any illicit drug use.

1 week before, this patient came to the small hospital that only have plain X-ray facilities. Patient came with abdominal pain and mild shortness of breath. Then the patient undergo chest X-Ray imaging which the result suggested Cardiomegaly and pneumoperitoneum in the upper right abdominal quadrant. This patient diagnosed with gastric perforation and then the patient referred to our hospital to get further treatment.

In our emergency room patient was afebrile with body temperature 36,5 °C, blood pressure 122/58 mmHg, pulse of 83 beats/minute regular, respiratory rate of 20 time/minute, and oxygen saturation level 99% on room air. His physical examination showed that he was cooperative, alert, and oriented to person, place, and time. His chest examination revealed that his lungs were clear to auscultation bilaterally, with no wheezing, no rhonchi, and no rales. His cardiovascular examination showed regular rate and rhythm, no murmurs, rubs, or gallops. His abdomen was soft, nontender, nondistended, no hepatosplenomegaly, normal bowel sounds, no muscle guarding with tympanic percussion tones.

From the Cardiology team, electrocardiography performed to confirm any cardiac problem from this patient and showed there was no abnormality on the ECG.

**Fig. 1:**
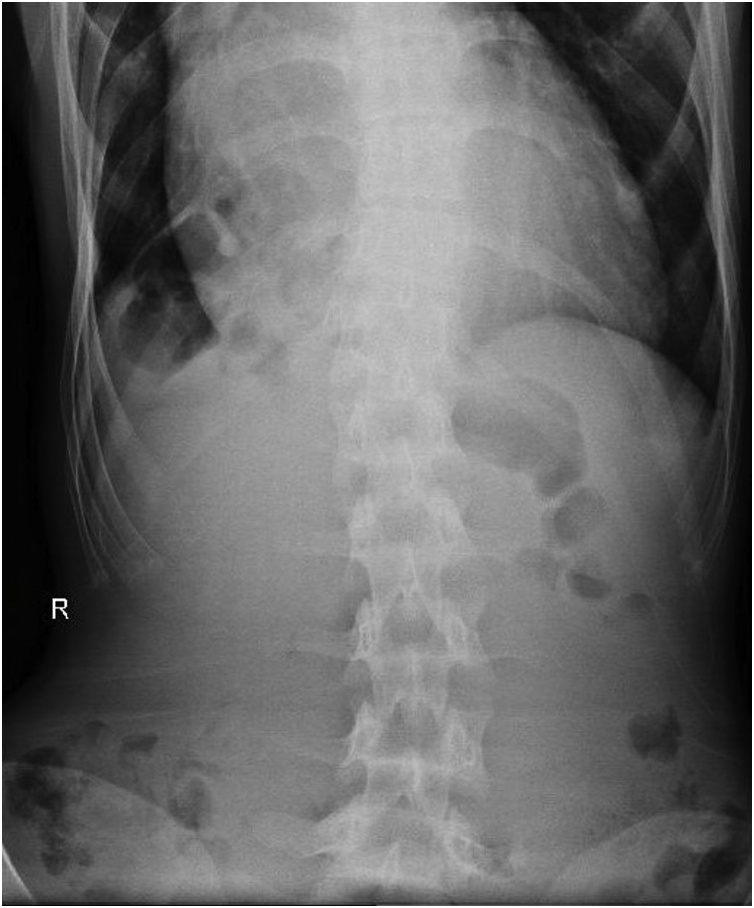
Plain abdominal radiograph suggesting the presence of subdiaphragmatic air.

**Fig. 2:**
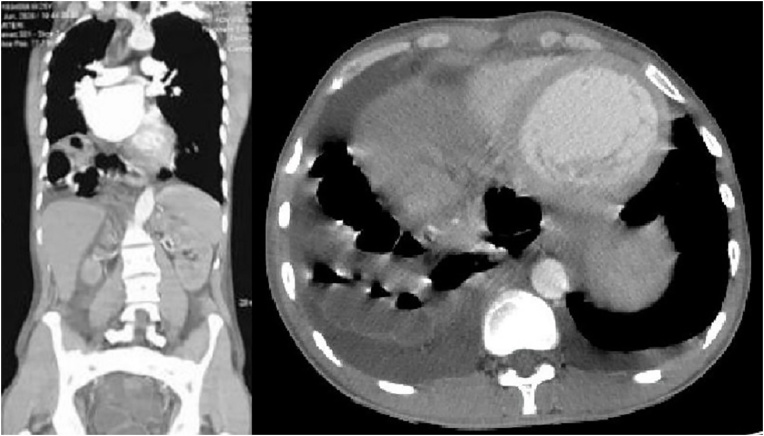
Abdominal CT-Scan showing colonic loop present between the right hemidiapraghma and liver.

Laboratory investigations was obtained and showing normal results. A plain abdominal x-ray was obtained (Fig. 1). It suggested the presence of an air-filled bowel tract within the right subphrenic space and the present of a cardiomegaly (Fig. 1). Abdominal computed tomography (Fig. 2) suggested colonic loop present between the right hemidiapraghma and liver, with absence of abdominal free-air that confirming isolated pseudopneumoperitoneum, due to colonic interposition between the liver and diaphragm.

In this case, the patient was treated conservatively with bed rest, analgesia and intravenous fluids. Decompression with nasogastric tube was already performed by the previous hospital because there was suspicious hollow viscus perforation although the patient was not showing any sign of gastrointestinal obstruction or peritonitis. We conclude that no surgical intervention necessary in this case. This patient discharged home 3 days after observation from the surgery and cardiology team

On outpatient follow-up 1 week post discharge, this patient showing no sign of abdominal discomfort or any sign of obstruction.

## 3 Discussion

Greek radiologist in 1910 Demetrius Chilaiditi, first described a radiographic finding of radiolucency in the subdiaphragmatic space as a result of colonic interposition is termed Chilaiditi's sign [1].

Symptoms that cause by Chilaidi’s sign is called Chilaiditi's syndrome, which primarily located in abdominal area, such as abdominal pain, anorexia, diarrhea, nausea, vomiting, bloating and constipation. Patients that have significant weightloss, redundant colon, high diaphragm, small liver, or absence of suspensory ligaments, all of which can be congenital or acquired are more likely to experienced Chilaiditi’s syndrome [2,4,5].

The differential diagnosis should consider primarily every potential cause of perforation of the abdominal hollow viscus. It can mislead physicians or surgeons to diagnosed it with diaphragmatic hernia, subdiaphragmatic abscess, bowel perforation, infected hydatid cyst and liver tumour possibly leading to unnecessary exploratory laparotomies [6,7].

The diagnosis is made primarily through imaging, such as plain chest or abdominal X-rays which may result in air filled bowel tract can suggest colonic interposition. But chest and abdominal X-rays are not as sensitive for the diagnosis as CT scans for the modality of choice [5,6,8].

In most cases nearly all patients are successfully managed by conservative treatment with bed rest, intravenous fluids and bowel decompression playing a significant role in alleviating the symptoms. Only occasionally, patients with recurrent presentation or evidence of vascular sufferance of the interposed bowel tract are offered surgical management [5,6].

Surgical treatment is reserved for patients whose symptoms do not resolve with conservative management or for suspicion of a complication such as ischemia or perforation. Surgical options may be performed using open, laparoscopic or robotic surgery with a variety of procedures possible to correct the interposition range from resection of the involved part of the colon (right hemicolectomy) or fixation of the liver to the abdominal wall to obliterate the potential space and prevent colonic displacement [3,5].

## 4 Conclusion

Until nowadays Chilaiditi’s Syndrome often misdiagnosed with hollow viscus perforation because the pseudopneumoperitoneum present in the plain X-Rays examination which can lead to unnecessary surgical procedure. Its important to understand the unique characteristic of the sign, symptomps and findings of Chilaiditi’s Syndrome. We suggest that conservative treatment is the best option of treatment for Chilaiditi’s Syndrome without any complication.

## Declaration of Competing Interest

The authors report no declarations of interest.

## Funding

The authors declare that this study had no funding source.

## Ethical approval

The informed consent form was declared that patient data or samples will be used for educational or research purposes. Our institutional review board also do not provide an ethical approval in the form of case report.

## Consent

Written informed consent was obtained from the patient for publication of this case report and accompanying images. A copy of the written consent is available for review by the Editor-in-Chief of this journal on request.

## Author contribution

Imam Sofii conceived the study. Imam Sofii, Sumadi Lukman Anwar, and Zakariya Aji Parminto drafted the manuscript. Imam Sofii and Sumadi Lukman Anwar revised the manuscript for intellectual contents. Imam Sofii, Sumadi Lukman Anwar, and Zakariya Aji Parminto facilitated all important tasks in the study.

## Registration of research studies

Not applicable.

## Guarantor

Imam Sofii.

## Provenance and peer review

Not commissioned, externally peer-reviewed.
